# Legal Notes for Institutional Workers

**Published:** 1913-10-04

**Authors:** 


### LEGAL NOTES FOR INSTITUTIONAL WORKERS.

### An Advance in Medical Jurisprudence.

The decision reported as *D.* v. *D.*, 29 T.L.R. 765, is such a distinct point of advance in the law as to nullity of marriage on the ground of refusal to consummate that it should be brought fully to the notice of all in any way interested in medical jurisprudence. The petition was presented at the instance of the husband, who craved dissolution of the marriage because of the wife's persistent refusal to consummate it. The spouses were forty-one and thirty-three years of age respectively. The husband was medically examined and found to be in every way normal; the wife refused to undergo medical examination; and she did not defend the suit. A letter from her was produced in which she reiterated her refusal, and bluntly stated that she did not want to have any children. The husband also deposed on oath to these and other relevant facts, and then his counsel moved the Court to infer from the facts that the wife was suffering from a physical incapacity or abnormality which justified them declaring the marriage null. It was in dealing with this motion that the President of the Divorce Court gave a straightforward deliverance on this class of case which has cut away much of the legal formality and fiction hitherto attending it. Why, he inquired, should he be asked to draw an inference of physical incapacity in this wife, when the evidence did not disclose anything of the sort, and the law presumed the opposite even, for did not Lord Stowell say: "A person need not be a physiologist to know how rarely an organ of the body is defective for the purposes of our nature. Malformation is not common in our sex, and perhaps it is still more uncommon in the other"? These queries led to the President investigating the earlier law and the progress of recent decisions, and, while he found that the cases disclosed a gradually developing legal opinion, his own judgment in this case is a further step in that development.

### The Old Precedent to Nullity.

In old days it was a necessary precedent to a nullity suit on the ground above referred to that the parties had cohabited for three years, so as to guard against any non-consummation being due to the natural coyness of the wife. That requirement was departed from, and action came to be allowed where the cohabitation had only lasted for a very short period. Then, again, the formal style of pleading in such cases stated that the defendant was "incurable by art or skill and will so appear on inspection," and to prove that allegation it was necessary to establish that the wife had been medically inspected and found to be the subject of some incurable malformation, and that partly explained the dicta in some cases to the effect that mere refusal to consummate was not enough, but there must be a medical inspection disclosing some incurable malformation. That rule also came to be toned down, and in cases where there was simply evidence of refusal to consummate the Court rather than refuse relief to the petitioning spouse acted on the legal fiction of inferring incapacity.

### Refusal or Incapacity as a Ground for Dissolution.

In one or two recent cases there were signs that the Judges were not satisfied with this theory, and now in the case we are discussing it has been quite discarded. The learned President refused to infer from the evidence put before him, the main points of which we have given above, that the wife was the subject of any incapacity; he did not in fact believe that any such existed, but found that the wife's refusal was wilful, determined, and steadfast. Did that in law entitle the husband to relief? The Court held that it did, and laid down, for the first time in express terms, that marriage implied the will as well as the ability to consummate, and was therefore voidable if the capacity or willingness to consummate was absent. Everyone will agree that to face and decide a straight issue like that was the best course. At the same time the judgment opens roads to dissolution of marriage which were closed so long as the old doctrine requiring that physical incapacity be proved either directly or inferentially held the field.

